# Process evaluation of a supportive intervention targeting social isolation among older people in Danish senior centres: Explanatory factors of implementation failure

**DOI:** 10.1371/journal.pone.0341550

**Published:** 2026-01-30

**Authors:** Sofie Langergaard, Rhiannon Evans, Jane Andreasen, Kirsten Schultz Petersen, Charlotte Overgaard

**Affiliations:** 1 Department of Health Science and Technology, Faculty of Medicine, Aalborg University, Gistrup, Denmark; 2 Clinical Nursing Research Unit Aalborg University Hospital, Aalborg, Denmark; 3 DECIPHer, School of Social Sciences, Cardiff University, Cardiff, United Kingdom; 4 Aalborg Centre of Health and Rehabilitation, Aalborg Municipality, Aalborg, Denmark; 5 The Unit of Health Promotion, Department of Public Health, Faculty of Health Sciences, University of Southern Denmark, Esbjerg, Denmark; Taipei Medical University, TAIWAN

## Abstract

This article explores the implementation of a supportive intervention in Danish municipal senior centres targeting social isolation among older people. The intervention, implemented between April 2022 and April 2023, comprised three key components: a start conversation for all new users; an assigned “buddy” among existing users; and monthly follow-up conversations. Skills development workshops for staff members were held prior to implementation of the intervention. The feasibility evaluation revealed concerns about the intervention implementation. This study describes the low level of implementation and explanatory factors contributing to the failure. We conducted a process evaluation as part of a feasibility evaluation of the intervention. The intervention was implemented in three municipal senior centres, ten senior centre staff members and 18 senior centre users participated. Data collection involved 23 semi-structured interviews with users and staff. Thematic analysis was conducted. Results are presented in two parts: 1) Overview of implemented components showing a low degree of fidelity in implementation, 2) Explanatory factors influencing implementation. The three factors identified were: A “too” systematic approach; Navigating frailty; and Lack of integration. These factors resulted in challenges recruiting participants and issues with performing some of the intervention elements. This evaluation provides insights into delivering interventions in municipal senior centres, emphasising explanatory factors to avoid implementation failures. The findings can support future development of contextually responsive interventions that can function as intended when delivered in real-world settings.

## Introduction

Social isolation among older people poses a significant challenge, contributing to adverse health outcomes, including loneliness, deterioration of physical health, poor quality of life and increased risk in mortality [1–3]. The risk of experiencing social isolation threatens the human need for belongingness [4] and is exacerbated for older people with physical and/or psychosocial frailties, since access to social communities can become limited [5–8]. Frailty, as conceptualised by Gobbens et al. [9], is a multidimensional phenomenon involving physical, psychological, and social components. The integral conceptual model of frailty highlights how these dimensions interact, emphasising that frailty is not solely a physical condition but also includes components such as emotional well-being and social relationships [9]. Understanding and addressing challenges related to frailties can possibly help bridge gaps in social engagement among older people, reducing their risk of experiencing social isolation. This resonates strongly with the Sustainable Development Goals (SDGs), particularly SDG 3, Good Health and Well-being, and SDG 10, Reduced Inequality, which aim to promote health, reduce inequalities, and foster social inclusion for all [10].

In a Danish context, local municipal senior centres have been identified as potential venues addressing social isolation [11]. These centres are publicly funded and offer recreational activities with a creative, physical and/or social focus, allowing individuals above the age of 65 to participate, without referral or registration. The centres are situated in local areas and function independently from other institutions such as nursing homes, health centres etc., and users are offered driving service at a small fee if necessary. The activities in each centre can be arranged by staff members or by the users themselves, and users can attend the activities at a low or no cost.

While numerous interventions have been implemented to address social isolation among older people and encourage socialisation with peers, such as group activities, network cafés and exercise classes, the effectiveness of these interventions has been limited, and few studies have been undertaken to explain why [12–21]. Danish municipal senior centres have previously been documented to be promising arenas for health promotion and disease prevention [11]. However, existing evidence on interventions targeting social isolation does not specifically address the potential of senior centres or their role in supporting the health and well-being of older people with frailties. In general, there is a paucity of intervention research and the impact of senior centres on the health and well-being of older people is significantly under-researched. To harness their potential, we developed a new intervention to support older people in becoming part of social communities and reduce social isolation which was implemented in a large Danish municipality [22].

We developed the intervention during the period April 2021-April 2022 adhering to the INDEX guidance [23] and followed a theory-driven approach. The INDEX guidance suggests a framework of 11 actions for intervention development (e.g., drawing on social theories, reviewing existing research, and articulating programme theory), which were followed closely during the development process. The associated programme theory is presented in S1 File Programme Theory. For further details on the intervention’s theory of change described in the programme theory we refer to the published development process [22].

The intervention comprised three key components:

*Start Conversations*: Within three weeks of a new users first visit, senior centre staff systematically invited new users for a start conversation, focusing on physical, mental, or social frailty factors [24], general health [25], and contact with other people [26]. The conversation addressed 50 questions, and the staff members entered the users’ responses in the software system REDCap (v. 14.0.16) by use of an iPad.

*Assignment of a “Buddy”*: Based on the conversation, a new user was paired with an existing senior centre user, who received guidance on supporting the newcomer.

*Follow-up Conversations*: Monthly conversations between staff and new users to identify any issues the new user might experience either with being new or with the “buddy”-companionship.

As part of the implementation activities, all staff members and managers attended two half day skills development workshops focusing on 1) The rationale for the intervention and its key elements, and 2) Being part of a research project and performing the data collection in REDCap.

This article reports data from the process evaluation considering the implementation of the intervention in municipal senior centres. It addressed the following research questions:

Was the intervention delivered with fidelity?

What factors explain the intervention delivery?

## Methods

This study was designed as part of a process evaluation focusing on assessing the feasibility of the developed intervention targeting social isolation among older people in the context of Danish senior centres. The process evaluation was designed with inspiration from the guidance for process evaluation of complex interventions by Moore et al. [27]. This widely acknowledged guidance has informed the UK Medical Research Council framework on developing and evaluating complex interventions [28,29], and was applied for this study, as it provides a structured approach to understanding how and why interventions work (or don’t), by evaluating components such as fidelity, implementation, and context. Focusing on the inadequacies of the intervention’s implementation, this study evaluates on the fidelity to which the intervention was performed, what was implemented and how, along with seeking explanatory factors to learn from the possible failures of the implementation. During the development process of the intervention, the CICI framework (Context and Implementation of Complex Interventions framework) [30] was used to address and plan the implementation of the intervention, taking important dimensions into account. Therefore, this framework was also used in the exploration and analysis of the findings of the evaluation. The article adheres to the reporting guideline COREQ (S3 File COREQ reporting guideline).

### Setting

The intervention was implemented, and feasibility tested in three senior centres within the Municipality of Aalborg, selected through a collaborative process involving senior centre management, who also contributed to the development of the intervention. This setting likewise provided the basis for the process evaluation, which focused on assessing the implementation of the intervention within these centres. Due to substantial differences in size, staffing structures and organisational characteristics across Danish senior centres, even limited descriptive details (e.g., number of staff or professional roles) could risk compromising anonymity. To protect the confidentiality of both staff and centres, such contextual data have therefore not been included. Prior to the intervention being implemented in the three senior centre informational materials were handed out to all staff members and users participating, including ethical considerations and information on the research team.

### Data collection for process evaluation

Data was collected for each of the research questions using different data collection methods. Data was collected by the first author (MSc and PhD student), who has worked with intervention research throughout the past four years, including performing interviews. A previous study in the context of senior centres had established a trusting relationship between researcher and participants, since management, most staff and many users had either met the research team or been part of previous study [11].

#### Assessing fidelity.

To address the first research question on the level of fidelity to which the intervention was delivered, what was implemented and how [31], quantitative data on the intervention delivery was collected to monitor and assess the fidelity over the period April 2022 to April 2023. We drew on the framework by Carroll et al. [31], which defines fidelity as a multi-dimensional construct. Specifically, we considered core elements such as adherence to the intervention protocol, dose delivered, and quality of delivery, while also recognising the moderating influence of contextual and participant-related factors. The quantitative data was questionnaire-based and managed and controlled through an electronical setup in REDCap (a secure web-based system for managing online surveys and data. Senior centre staff members were to collect background data and survey data from the participants and register this in REDCap. The collection of data was monitored weekly by the first author, who also collected and wrote down supplementary data relevant to assess intervention fidelity, obtained through informal situations, such as morning meetings with the staff members of the included centres (attended biweekly the first three months and then monthly), phone calls between the researchers and staff members, and casual meetings with users. An overview of the delivery of the intervention is presented in Table 2 along with participants’ individual level of frailty estimated by use of the TFI questionnaire [24]. A score above five indicate that the participant is experiencing physical and/or psychosocial frailty [24]. For anonymity, the only participant data reported is civil status and educational level. Data on frequency of user visits were not possible to collect, nor was data on the total number of new users in proportion to users invited into the intervention. Generally, no registration of user visits is allowed according to Danish legislation for senior centres.

#### Explanatory factors of implementation.

Seeking explanatory factors to understand the implementation and fidelity of intervention delivery, as the second research question of this study, all users and staff members participating in the intervention were invited to interviews following a purposive sampling [32]. Among the senior centre users participating in the intervention 13 out of 18 accepted to take part in semi-structured interviews. All 10 full-time staff members working in the three senior centres where the intervention took place consented to interview. Part-time staff varied widely in their availability and assigned tasks, and many were not in positions that allowed active involvement in the intervention. To ensure consistency across centres and support a coherent implementation process, the implementation was therefore anchored primarily with full-time staff. There were 23 interviews total conducted in the period December 2022 to March 2023. Staff represented a diversity of professions and educational backgrounds such as: social and health care assistants, nutritional assistant, occupational counsellor, or unskilled workers, but no details are shown to protect the anonymity of the staff members.

The location for each interview was chosen by the interviewee. All staff interviews were held at the senior centre they worked at, and the interviews with users were held either in the senior centre they attend or in their homes, based on their own choice. Interviews were semi-structured and conducted using an interview guide (Main topics of the interviews is seen in S2 File Interview guide). Interviews were held towards the end of intervention delivery or in the immediate period after completion (over a period of three months in total). They had a duration of 40 min in average for staff interviews and 37 min. in average for user interviews.

### Data analysis

Interview data was transcribed and handled in the software NVivo (v.12) by the first author, where it was coded following the concept of thematic analysis [33]. The initial part of the analysis was guided by the CICI framework [30], that also inspired the initial intervention development, and data was sorted accordingly.

Subsequently, a more inductive approach was employed to read and code each pre-sorted section. The steps of the analysis are described in detail in Table 1.

**Table 1:** Execution of the six phases of thematic analysis.

Phase	Description of the process
**1. Familiarising yourself with your data**	Interviews were transcribed and read thoroughly. A preliminary understanding of the interview data was written down.
**2. Generating initial codes**	Sentences and smaller passages were systematically coded using the CICI framework’s [30] intervention domains: theory, process, strategy, and agents as well as the intervention’s overall fidelity, dose, and reach. Thereby repeated patterns about the implementation were identified and sorted.
**3. Searching for themes**	Themes were formed reading through the codes looking at what appeared as major issues in the implementation of the intervention.
**4. Reviewing themes**	Reading through the codes and themes over several rounds to verify the themes. These were then compared to the preliminary understanding of the interview data.
**5. Defining and naming themes**	The phases 5 and 6 were performed concurrently and the defining and naming of the themes was settled through writing out the themes.
**6. Producing the report**	The reporting of the analysis was produced in a dynamic process starting already in phase 5.

### Ethical considerations

In compliance with Danish regulations, studies not involving biological materials does not require approval from an ethics committee but rely solely on obtainment of informed consent from participants [34]. The study adhered to the European Code of Conduct for research integrity [35] and data management followed both Danish and European General Data Protection Regulation [36,37]. The study was approved by Aalborg University and the municipality of Aalborg. In line with the Helsinki Declaration [38], participants were thoroughly informed about the study’s purpose, data protection, and voluntary participation in the interviews with the right to withdraw consent at any time, complete with signed written consent from all participants.

## Results

The presentation of the results of the process evaluation is organised in two sections. The first section, based on the quantitative data collected, addresses the intervention’s implementation and fidelity by presenting an overview of the intended implementation, including an assessment of actual delivery in practice, which was considered to be inadequate (Table 2). Overall, the implementation did not go as planned and intervention fidelity was low. The second section therefore addresses the explanatory factors that help to understand this implementation failure. This result section is based on the interview data. Statements from staff members dominate this section, as they were the ones with direct experience of the implementation, and therefore, the most able to provide explanations to why the implementation failed.

**Table 2:** Intervention participants and elements: Received components and descriptive information.

Centre	ID	Intervention elements									Consented to interview	Participant details		
		Start conversation	TFI score	Assigned “buddy”	Follow-up 1	Follow-up 2	Follow-up 3	Follow-up 4	Follow-up 5	End conversation		Marital status	Age	Educational level
**C1** Urban	1.1	✓	6			✓	✓		✓	✓	Yes	Separated/divorced	65	Trained craftsperson
	1.2	✓	9	✓	✓	✓	✓	✓	✓	✓	Yes	Separated/divorced	85	Trained craftsperson
	1.3	✓	4	✓	✓	✓	✓	✓	✓	✓	Yes	Widow/widower	84	Medium higher education
	1.4	✓	6	(✓)		✓				✓	Yes	Separated/divorced	68	Elementary school
	1.5	✓	5	✓		✓	✓	✓	✓	✓	Yes	Unmarried	85	Medium higher education
	1.6	✓	6								No	Widow/widower	85	Medium higher education
	1.7	✓	4	(✓)		✓	✓				No	Married/cohabiting	85	Trained craftsperson
**C2** Urban	2.1	✓	1			✓		✓	✓	✓	Yes	Married/cohabiting	71	Short higher education
	2.2	✓	3		✓	✓		✓	✓	✓	Yes	Married/cohabiting	77	High school
	2.3	✓	2		✓	✓	✓	✓	✓		No	Married/cohabiting	83	Medium higher education
	2.4	✓	7	✓	✓	✓	✓	✓	✓	✓	Yes	Separated/divorced	72	Medium higher education
	2.5	✓	6		✓	✓	✓		✓	✓	Yes	Separated/divorced	78	Trained craftsperson
**C3** Rural	3.1	✓	2	✓	✓		✓	✓		✓	Yes	Widow/widower	81	Trained craftsperson
	3.2†	✓	12	‡							No	Widow/widower	87	Trained craftsperson
	3.3	✓	9	✓		✓	✓		✓	✓	Yes	Separated/divorced	67	Trained craftsperson
	3.4	✓	5				✓	✓		✓	Yes	Married/cohabiting	69	Medium higher education
	3.5	✓	2	✓	✓	✓				✓	No	Married/cohabiting	75	Trained craftsperson
	3.6	✓	9	‡							Yes	Married/cohabiting	65	Trained craftsperson

† Participant died during the intervention period.

‡ Participant was not told about the possibility of getting a “buddy”.

(✓) Accepted the offer of getting a “buddy”, but staff were not able to find a match.

### Overview of implementation fidelity

Table 2 present each element of the intervention that were to be delivered and whether it was or not (marked grey). Furthermore, Table 2 shows participants’ frailty score (TFI score), where a score above five is considered to indicate that the participant is experiencing physical and/or psychosocial frailty. In the right part of Table 2 (marked white) is information on whether they consented to interview or not, and limited data about each participant, also included.

Overall, the intervention was not delivered as intended and fidelity is therefore considered to be low. The recruitment of participants failed to reach the anticipated number of minimum 25 in the scheduled time-period of two and a half months. However, this anticipation was challenging as it was based solely on estimates provided by the senior centres management, given that according to Danish legislation, it is prohibited for senior centres to collect or record any information about their users. We set the minimum number of 25 new users for the recruitment period, as this was estimated to provide sufficient insight into the implementation process to enable a meaningful evaluation of the intervention [27]. In response to this shortfall, two strategic actions were undertaken to heighten participant enrolment: firstly, an extension of the recruitment period, first to four then six months, and secondly, allowing staff members to recruit users from the senior centres who had commenced their attendance within the previous four months, thereby still meeting the criterion of being deemed new in a senior centre setting. However, recruitment of intervention participants still failed, ending on 18 in total over the course of six months. All new users, included in the intervention, did receive a start conversation, as this set off their participation in the intervention, without this component, the new user could not be considered an intervention participant. Assignment of a “buddy” to each new user and performing the follow-up conversations were also not conducted as prescribed. Nine of the 18 participants accepted a “buddy”. With two of the nine, staff failed to find a matching “buddy”, therefore only seven ended up getting a “buddy”. Prior to each scheduled follow-up conversation, researchers would write to the responsible staff members a couple of weeks in advance. Despite this, the execution of follow-up conversations failed to occur.

### Explanatory factors

As the implementation of the intervention for this feasibility study failed, and the intervention was delivered with low fidelity, understanding these challenges became the main focus of the analysis of the interviews. Three explanatory themes emerged to operationalise the implementation failure: Theme 1: *A “too” systematic approach*. Theme 2: *Navigating frailty*. Theme 3: *Lack of integration*.

#### A “too” systematic approach.

Over the course of the implementation period staff members experienced the systematism of the intervention challenging. The intervention was intentionally developed to resemble the usual practice usual of senior centre staff members, but more systematic across the centres of the municipality. This should allow all new users to get the same experience when being new in a municipal senior centre and make it easier for staff members to identify, who needed more support than others, allowing for them to allocate the time where most needed. The systematism of welcoming all new users the same way proved harder for the staff members than initially expected, both by them and the research team. Some staff members began to perceive the systematism as negative during the intervention period and even came to dislike it:

“I really don’t like it. I really don’t like that it has to be systematised. Especially because we don’t have an iPad, and we don’t have [registration system], and people are so different.” Staff member 7

This indicated that the intervention could be affected by a lack of system fit, and when having to perform the intervention it became a big task that none of the staff members really wanted to do, which resulted in them leaving it, hoping another staff member would take care of it:

“It wasn’t because I didn’t find the project interesting and it wasn’t because I thought, ‘You can’t do that.’ I didn’t think that finding a “buddy” was impossible; it’s just that it seemed time-consuming, I believe. But... no, it’s just not for me. I will just come up with some excuses, like ‘you guys go ahead’.” Staff member 8

The systematism of the intervention especially proved difficult when it came to the monthly follow-up conversation, resulting in staff members not performing all of these. This was not only because they felt it to be time-consuming or because they made excuses for not performing the follow-up conversations, but seemingly also because of the frequency:

“Some come here every day, some come here twice a week. And for those who come twice a week, it might make sense to be asked, as opposed to those who come every day of the week.” Staff member 2

Performing the follow-up conversations following the systematism could feel less meaningful for some staff members because it did not meet the needs of every new user, and therefore an inclination to adjust the frequency of the follow-up conversation to each user arose, affecting the fidelity of the implementation of the intervention. Among the participants the new follow-up conversations were perceived and embraced very differently with a few having a distinct positive experience:

“I think it has been nice. We just sat and had a pleasant chat for a while. Of course, other things came up as well. Naturally. It was good that we followed up on something from last time, went over things, and checked them off. I’ve felt good about it every time. It has probably meant more to me than I initially expected.” Participant 3.3

Although most felt more or less indifferent about the follow-up conversations:

“We had to after all. And it was nice enough, but that was that.” Participant 3.4

The highly varied reception of the follow-up conversations among the participating users supports the staff members’ perception that users had significantly different needs regarding these conversations.

Another factor was the use of an iPad and the data entry in the electronic REDCap system during the start conversation. Some staff members were unfamiliar with digital devises and found the technical use of an iPad hard, despite having received training prior to the project start:

“I’m not very skilled with computer stuff like that. So, […] that has sort of shifted to [another colleague].” Staff member 10

Not being comfortable with the technology made some staff members withdraw from participating in the tasks of the intervention, resulting in leaving it to their colleagues.

#### Navigating frailty.

While implementing the intervention, staff members encountered challenges in navigating the varying levels of frailty among participants, which consequently impacted elements of the intervention, such as participant recruitment and start conversations, resulting in a deviation from the expected fidelity and thereby negatively affecting implementation.

The staff members expressed the overall perception, that results of the frailty screenings performed during the start conversations generally matched their preconceived notions of the psychological, social and/or physical frailty of the new user prior to screening. However, some also reported being surprised by the result of the screening:

“Yes, there was one I was quite surprised that, in fact, he was so capable and didn’t have more difficulties than that. But it’s probably a bit of a question about who he was as a person because, in my opinion, he has many challenges [...] but he doesn’t seem to think he has any difficulties [...], and I was surprised that he stands out the way he does, but he also has a great network and actively seeks out many things, so yes. And then there’s another one where I was also surprised how poorly she actually was.” Staff member 3

This and other examples from the data indicate, that screening for frailty in some cases deviated from the staff members’ intuitive assessment and could offer valuable insights into the health and well-being of the new senior centre user but only a few staff members took notice of the score. Most staff members seemed uncertain about the frailty score or did not feel it had any use for them and none of them used it actively when searching for a matching “buddy”, as intended:

“No, I don’t think so. Of course... I probably looked down on it for what it was, and then I thought, ‘Oh, I can’t... It’s not something I need for anything, that score thing’. That’s probably how I thought. And then I haven’t thought more about it. I haven’t.” Staff member 2

This sentiment was echoed by many staff members, indication a broader issue with implementing frailty screening as part of the start conversation. The uncertainty surrounding the utility of the frailty score suggests that the research team may not have sufficiently or clearly communicated its purpose, resulting in the fidelity of performing this part of the intervention being low.

In some cases, staff members self-assessed some new users as too frail for being invited to take part in the project, inattentive to or unaware of this going against the purpose of the intervention; that all should be offered a start conversation where their need for support could be assessed according to validated tools and questionnaires, giving all a chance to get the support needed.

“Yeah, well, there have probably been a couple we specifically didn’t ask because we’ve had that discussion about whether it was relevant or not. There have been one or two we have excluded.” Staff member 4

This approach led to exclusion of potential participants particularly those perceived as frailer, due to concerns about their ability to fully engage with the project activities and the perceived burden of finding suitable ‘buddies’. This resulted in two new users not getting offered a “buddy” among the existing users (see Table 2), and staff members not performing this part with fidelity to the intervention’s overall purpose. As a staff member explained:

“Yes, they have been frail or... we had one who was simply impossible to find a “buddy” for because it was difficult to find someone who matched. Um, the user lived under some particular life conditions, you could say, so it was hard to find a “buddy” who could handle the task.” Staff member 3

The recruitment of participants was meant to occur the first or second time a new user came to a senior centre. Staff members were instructed to hand out informational materials and ask all new users to participate in a start conversation at the earliest convenient time. However, this approach proved daunting for the staff members, who felt many new users could get overwhelmed by this initiative:

“I simply think that we scare people away as well. Because phew’, ‘What do you want to use it for?’, ‘why this?’, right? I mean, ‘What is this about? That’s a strange thing to ask about’.” Staff member 6

In response to staff concerns, the protocol was adjusted allowing staff to delay the start conversation until approximately two weeks after a new users start. It does however reflect a misalignment of frailty since the intended prompt invitation for a start conversation was undertaken to engage and ensure adequate support for new users with frailties. Despite the adjustment, staff members generally still found it challenging to implement this intervention component, as it conflicted with their perception of older people with frailties’ readiness to commit to this at their initial visits to a senior centre.

The new users likewise undertook self-assessments of their own level of frailty and the relevance of being matched with a “buddy”. Some of the new users declined the offer of getting a ‘buddy’, with some explaining that they did not feel they needed one:

“And then, of course, she asked if I wanted a “buddy”... No, I didn’t think that was necessary. I believe I could manage on my own. No, I didn’t need that, no.” Participant 2.2

In some of these instances, staff members would agree, and the frailty score would be low, as in this case with Participant 2.2, who had a frailty score on 3, indicating that the user may be right about not needing support. Nevertheless, four new users declined being matched with a “buddy” although their frailty score was five or higher, and having a buddy, according to the underlying understanding of frailty in the intervention, could have been a support during the new users’ initial visits in a senior centre. When asked about this later during the individual interviews one of these users expressed regret for declining the ‘buddy’ as he had experienced being new in a senior centre as difficult, especially socially:

“I actually would [like to have accepted]. Yes, there are many things I would like to participate in, […] so, uh... I’m not that social, even though I have been, yes.” Participant 2.5

The main reason users gave for declining the offer of getting a ‘buddy’ was that they did not want to burden other people with the responsibility of taking care of them and/or like Participant 2.2, they felt, they could enter the social communities on their own, not needing additional support. This feedback suggest that the “buddy” offer was perceived as a special arrangement rather than a standard practice in the senior centre. The implementation thus did not successfully integrate the offer of a buddy as ‘usual practice’, invertedly stigmatising those who might have benefitted from additional support.

#### Lack of integration.

The intervention never became fully integrated as part the senior centre practice because of different barriers encountered. The usual practices of the senior centres did not allow for staff members to prioritise the tasks of the intervention. Staff members struggled to integrate the intervention components in their daily work:

“Yes, there’s no doubt that when you feel busy, this... it could easily be pushed a bit. It has certainly happened for me. I felt like I should solve the other tasks first, right? Because, after all, that was my job.” Staff member 1

This sentiment highlights a fundamental issue: the intervention remained an ancillary task rather than becoming embedded in the staff’s daily workflow. The need for prioritising the daily tasks negatively affected the implementation of the intervention, with the staff simply not delivering the intervention components with the intended fidelity. During the recruitment period of the intervention staff members informed the research team that they were inviting all new users to participate in the research project, but when we ended the recruitment period, ultimately lasting six months, staff members revealed that they did not ask all new users:

“No, I really don’t think we have. I think some have attempted it, but maybe also thought ‘ahh’ a bit [reluctant sound, indicating doubt].” Staff member 2

The need for prioritising their daily tasks made some staff members ask the senior centre management, about whether the tasks related to the intervention was ‘nice to’ or ‘need to’ perform:

“And when you mentioned that we should have this interview, I immediately wrote to [leader’s name], ‘Is this something we need to prioritise?’ and she replied, ‘Yes, you should,’ so I said, ‘Okay’.” Staff member 6

Despite this affirmation, the staff members kept feeling that the intervention was an addition to their daily tasks, causing the new systematic approach to never become fully disruptive of the usual system and therefore not reach the point where the overall potential could be determined or not.

The intervention was thought out to be performed by all staff members of each senior centre, with them working as a team, aiming for a full integration of the intervention. However, time constraints emerged as a critical factor negatively affecting the tasks linked to the intervention. In this context many wished for allocated time and maybe also allocated staff members, who had the responsibility for the tasks to be performed:

“‘And we’ll assign it to [two staff members], and each of you will find 6. You won’t have ‘that’, and you won’t have ‘that’ during this period because you’ll be running this project and have time for it.’ It would have been completely different instead of having it on all of us, and we’re all running around like headless chickens.” Staff member 8

To accommodate this issue, one of the senior centre’s staff members made a mutual understanding that only one of them would perform start and follow-up conversations, while the other staff members would help find matching “buddies”, when this occurred. This strategy however did not solve the time issue. Conversely, it led to other challenges in the staff group, as the staff member felt alone and somewhat overwhelmed with the tasks while the other colleagues felt guilty:

“I also feel that perhaps we let [colleague] down a bit, or what should one say? That it sort of hung on her, all these conversations. But it was also, I want to defend a bit, that it was something we did in between everything else.” Staff member 10

The adjustment did not yield the intended positive impact on the implementation and the intervention remained being delivered with a low degree of fidelity. One evident indication that the intervention failed as an integrated part of the daily workflow of staff members was that many staff members framed the intervention as assistance for a research study rather than a meaningful opportunity for the new senior centre user that they genuinely wanted to incorporate into their approach to welcoming new users in the senior centre:

“Yes, and then you sit there, ‘[name of researcher] also wants to know… it sounds a bit like what I just asked about’. Yes, yes, but then they answer to the best of their ability, yes.” Staff Member 3

Staff members talked about screening for frailty with distance as they generally came to see the intervention as something they were performing for others. In the same way they recruited participants with a certain distance, saying that help was needed for a research study. When participants were invited for participation, their main reason to consent to this study was that they wanted to help both the staff members at the senior centres and the research team:

”She had some papers that she said we could take a look at; whether it was something I might be interested in because there was someone at the university... So, I immediately said that I would be more than happy to, without knowing what it was about.” Participant 3.1

The systematic approach to welcoming new users was intended to be presented as an integrated part of the senior centres’ daily tasks by the staff members, not exhibited as an option both staff members and participants could refrain from. Based on their approach to recruitment and start- and follow-up conversations of the intervention, elucidating that this was mainly for research, integration was never achieved. This may be an important aspect of explaining the mechanisms at play and why the fidelity of intervention delivery was low.

## Discussion

In this study, an intervention targeting social isolation among older people in Danish municipal senior centres was implemented and assessed through a feasibility study with an integrated process evaluation. The intervention encompassed three key components: a start conversation for all new users, an assigned “buddy” among existing users, and monthly follow-up conversations between staff members and the new user. Before performing the intervention, skills development workshops were held for staff members. Fundamental challenges emerged during the feasibility test, leading to concerns about implementation success.

Assessment of implementation revealed insights into the challenges of real-world delivery. Implementation failure was evident in difficulties with participant recruitment, low acceptance of “buddies”, and issues with performing all follow-up conversations (Table 2). Experiencing challenges with implementation, explanatory factors were sought in the interview data. This was identified in the thematic analysis as the intervention having a “too” systematic approach and being affected by staff members having to navigate frailty, as well as a lack of integration of the intervention, overall indicating that staff members faced dilemmas in recruitment, participant eligibility and struggled to integrate the intervention into their daily tasks. Focusing on these explanatory factors operationalise the results to contribute a deeper understanding of the implementation failure, rather than simply stating whether it is performed with fidelity or not. Something that has been requested in researching complex interventions in health and social care among older people [39].

The intervention having a systematic approach aimed to welcome new users uniformly across all senior centres, contrary to the current approach, where each senior centre or each staff member would have their own approach. The staff members feeling that the approach was “too” systematic for them could reflect them not having attained adequate competences to perform the intervention, possibly needing more instructions than the two half-day skills workshops held prior the intervention beginning. This raises questions as to which extent the skills workshops succeeded in transferring the intended knowledge that was assessed to be necessary for staff members to perform the intervention. Knowledge transfer is a concept often used as an integral part of the implementation, where a developed framework can contribute with reflexions and guidance on how to ensure or improve the transfer of the knowledge needed for both those participating in or delivering interventions in healthcare [40,41]. Staff members in Danish municipal senior centres have very diverse educational and/or work-related backgrounds and therefore also very different prerequisites for understanding the aim of the intervention and why and how to perform it. This would have to be taken into consideration with future research in this area, where focussing on adequate transfer of knowledge is crucial.

When staff members had to navigate frailty, their different understandings became evident over the course of the intervention period. Frailty in research is an acknowledged and very explored concept and often referred to when dealing with research among an older population with vulnerabilities caused by aging and late-life constraints [8,42,43]. Many different approaches can be taken to frailty, and the understanding has developed throughout the years from focusing mainly on physical aspects of being frail to encompassing psychological and social aspects [9]. Frailty therefore becomes a complex state, where discourses of the understanding can arise. In the implementation of the intervention, staff members’ understanding varied within their own profession, and at the same time the different understanding would, in some cases, be contrary to the understanding attained by the research team. This caused staff members to exclude new users because of their frailties, going against the aim of the intervention. Staff members understanding frailty as a holistic concept, as described by Gobbens et al., 2012 [9], was never fully obtained, which could indicate that further instructions or collaborative activities could be necessary for further development of the intervention and future implementation of alike.

Lack of integration of the intervention marked itself as staff members continuously throughout the intervention period thinking of the intervention as additional tasks to perform in their already planned out timetables, rather than a part of their daily work. The intervention was not prioritised over usual practice, and these two were perceived as mutually exclusive. This raises questions about how people see new interventions and how to handle different perceptions of these. The understanding that intervention tasks could be comprehended as ‘nice to’ rather than ‘need to’ by those who are to deliver the intervention underlines an issue with the acceptability of the intervention. The theorisation and conceptualisation of acceptability of interventions [44] has taken up more space in the research over the last few years suggesting that we should focus on elaborating and asserting this perspective as a construct to intervention engagement and adherence along with process evaluation of implementation, mechanisms of change and context. Examining the acceptability of interventions could provide important knowledge to comprehend the potential impact and effectiveness in the future.

### Strengths and limitations

The intervention was developed using stakeholder involvement where representatives from senior centre users, staff members and management participated in designing the intervention in terms of activities, content, and timeframes [22]. However, when it came to implementing the intervention, the design was not applicated as intended. This could be attributable to the process, broadness, and techniques of the involvement, but since internationally acknowledged guidelines [23,28] were used in the development process, this could also be a question of looking more closely at the explanatory factors, also divided into individual- and system level explanatory factors, that could impact the implementation of the intervention beforehand [45]. Enhancing the system level focus of stakeholder involvement in intervention development could possibly reduce potential implementation challenges [45].

More detailed information about the organisational structure and staffing of the participating senior centres could have enriched the contextual understanding of how the intervention was implemented, for example by illustrating differences in staff capacity, task distribution, and organisational conditions that may have shaped implementation processes. However, due to the considerable variation between Danish senior centres in size and staff composition, reporting details such as FTE distribution or role allocation would have risked compromising the anonymity of staff and, by extension, the centres themselves. To safeguard confidentiality, we therefore refrained from including this level of detail, despite its potential analytical value. Also, while additional demographic information about senior centre users (e.g., gender, physical disability, or use of hearing aids) could have offered further nuance, it was not essential for addressing the study’s primary aim. The focus of this paper is on the implementation process and the organisational and staff-related factors that shaped it, rather than on user-level outcomes. Furthermore, participating service users represented a broad range of ages, health statuses, and levels of frailty, and frailty screening provided an overall assessment of their physical and psychosocial resources, and therefore we do not consider unreported user characteristics to have influenced the findings or the interpretation of the implementation challenges.

The implementation of the intervention could, in part, have been affected by the researchers’ status as external to the senior centres. While the establishment of a prior relation was a strength allowing for better understanding of the context, this familiarity may not have fully mitigated the challenges of being perceived as an “outsider” [46]. This position could have been seen as interfering or invasive, potentially leading to some resistance or distance. However, it is important to note that the strong support of the senior centre management could likely have helped to smooth the process and reduce potential friction caused by the external character of the researchers. The research team has extensive experience in qualitative health service research and in working with older people in community settings. All hold a health science background and are trained in qualitative interviewing techniques. Being aware of and to account for own potential biases and assumptions, stemming from professional perspectives, and previous experience in the field, the team continuously engaged in reflexive discussions to reflect on their positionalities and to enable an open and impartial approach to the data collection and analysis.

This process evaluation focussed on the implementation issues of the intervention rather than the outcomes of the intervention since the intervention never was fully disruptive of the complex system of municipal senior centres [45]. In this situation the intervention’s theory of change has not been tested fully, nonetheless the results provide insights into the theoretical underpinnings of the intervention and suggest a potential for redefining and redesigning the intervention for a better context fit and overall effectiveness. The uncertainty about the intervention’s theory of change, operationalised in the programme theory of the intervention (S1 File Programme theory), makes it necessary to perform another feasibility test for careful consideration of the intervention’s real-world applicability and to mitigate the implementation barriers encountered in this study. Therefore, uncertainties about whether the intervention’s theory of change itself may have contributed to the implementation issues still stands.

### Implications for practice and future research

By exploring senior centres, a relatively unknown field of research, this article sheds light on the complex and diverse setting municipal senior centres have shown to be and moreover uncover possible approaches and challenges to future interventions in this and similar settings. Municipal senior centres remain important arenas for addressing health among older people, however the possibilities for making use of this potential have yet to be tested thoroughly and effectively. Conducting this initial research project within the Danish senior centre setting posed challenges in terms of for example, estimating the potential participant recruitment and how many users we would be able to recruit, since there was no available data or registrations on the influx of older people to inform the recruitment process. With the knowledge and experience from this study, future research will be better informed in this area.

## Conclusion

The article contributes valuable insights for the implementation of supportive interventions in municipal senior centres and possibly other municipal settings alike, as well as underscores the significance of understanding explanatory factors for implementation failure when developing and implementing interventions.

The implementation of the intervention was delivered with low fidelity, and three main explanatory factors were found to affect the implementation. The systematic approach proved more challenging than expected for staff members and with frailty among older people being a dynamic and complicated state, staff members’ thoughts on who was eligible for the intervention and who were not affected the recruitment. The intervention never became fully integrated in staff members’ daily work.

The discovered insights hold the potential to inform future development and feasibility testing of interventions aimed at addressing social isolation among older people and the implementation failures discovered offer valuable guidance for refining future research and practice interventions and programmes.

## Supporting information

S1 File Programme theory. (DOCX)

S2 File Interview guide. (DOCX)

S3 File COREQ reporting guideline. (DOCX)
