# Supplementary figures and images for: Process evaluation of a supportive intervention targeting social isolation among older people in Danish senior centres: Explanatory factors of implementation failure
Source: PLoS One. 2026 Jan 30;21(1):e0341550. doi: 10.1371/journal.pone.0341550 (PMC12858015; doi:10.1371/journal.pone.0341550)

Supporting Information 1


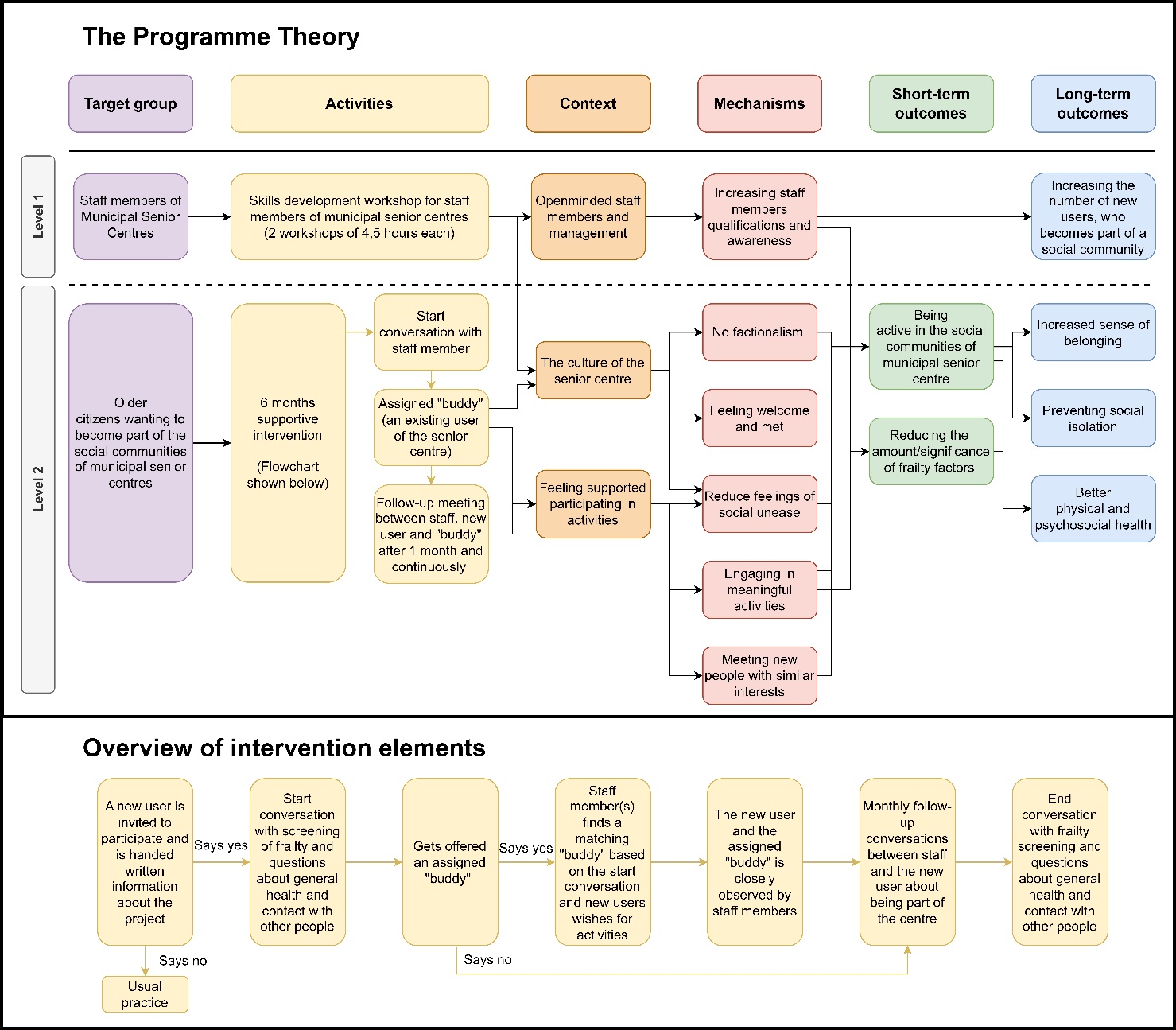

Supplement: S1 File — (DOCX) [file pone.0341550.s001.docx]
