# Supplementary material for: Process evaluation of a supportive intervention targeting social isolation among older people in Danish senior centres: Explanatory factors of implementation failure
Source: PLoS One. 2026 Jan 30;21(1):e0341550. doi: 10.1371/journal.pone.0341550 (PMC12858015; doi:10.1371/journal.pone.0341550)
Supplement: S2 File — (DOCX) [file pone.0341550.s002.docx]

Supporting Information 2

Main topics of interviews with senior centre staff members:

|  | Process Evaluation domain | Research questions |
| --- | --- | --- |
| Implementation | Training of staff members | 1: Did the introduction workshops cover what was needed?  2: How many staff members participated in the workshops?  3: Did staff members know what to do after the workshops? |
|  | Recruitment of participating users | 4: Were all new users asked to participate in the intervention?  If not:  - 4A: Why?  - 4B: Were there differences between the ones being recruited and the ones that wasn’t?  5: Any differences in who recruited new users and who didn’t? |
|  | Intervention components (conversation, buddy, follow-up) | 6: Were the three intervention components all delivered in each senior centre?  6A: If differences between the delivery of any of the components: which, how and why? |

|  | Process Evaluation domain | Research questions |
| --- | --- | --- |
| Mechanisms of impact | Responses to and interactions with the intervention | 1: How much has each staff member interacted with the intervention?  2: Have any of the components of the intervention required more work than others? |
|  | Mediators | 3: Did the mechanisms of the intervention work as intended?  4: Were any of the mechanisms recognisable during the intervention? |
|  | Unexpected pathways and consequences | 5: Did any unintended consequences arise from the intervention? Both positive and negative? |
|  | Process Evaluation domain | Research questions |
| Context | Contextual factors that shape how the intervention works | 1: Did each centre experience any contextual impact on the intervention?  2: Can we say anything about what “type” of centre the intervention suits best? If any. |

Main topics of interviews with senior centre users:

|  | Process Evaluation domain | Research questions |
| --- | --- | --- |
| Intervention | Invitation and participation | 1: How was the intervention presented to new users?  2: What were the invited participants motivation to participate in the intervention? |
|  | Intervention components (conversation, buddy, follow-up) | 3: Did the participants receive all three components of the intervention?  4: What was the experience of each component? |

|  | Process Evaluation domain | Research questions |
| --- | --- | --- |
| Mechanisms of impact | Mediators | 1: Did the mechanisms of the intervention work as intended?  2: Were any of the mechanisms recognisable during the intervention? |
|  | Outcomes | 3: Were the outcomes of the intervention reached among the users?  Both short-term and long-term. |
|  | Unexpected pathways and consequences | 5: Did any unintended consequences arise from the intervention? Both positive and negative? |

|  | Process Evaluation domain | Research questions |
| --- | --- | --- |
| Context | Contextual factors that shape how the intervention works | 1: Did new users experience any contextual impact during their start in the senior centre? |
